# Catalytically active prokaryotic Argonautes employ phospholipase D family proteins to strengthen immunity against different genetic invaders

**DOI:** 10.1002/mlf2.12138

**Published:** 2024-09-04

**Authors:** Feiyue Cheng, Aici Wu, Zhihua Li, Jing Xu, Xifeng Cao, Haiying Yu, Zhenquan Liu, Rui Wang, Wenyuan Han, Hua Xiang, Ming Li

**Affiliations:** ^1^ Department of Microbial Physiological & Metabolic Engineering, State Key Laboratory of Microbial Resources, Institute of Microbiology Chinese Academy of Sciences Beijing China; ^2^ College of Life Science University of Chinese Academy of Sciences Beijing China; ^3^ State Key Laboratory of Microbial Resources, Institute of Microbiology, Chinese Academy of Sciences Beijing China; ^4^ State Key Laboratory of Agricultural Microbiology and College of Life Science and Technology, Hubei Hongshan Laboratory Huazhong Agricultural University Wuhan China

**Keywords:** Argonaute, DNA interference, genome editing, phage defense, PLD protein

## Abstract

Prokaryotic Argonautes (pAgos) provide bacteria and archaea with immunity against plasmids and viruses. Catalytically active pAgos utilize short oligonucleotides as guides to directly cleave foreign nucleic acids, while inactive pAgos lacking catalytic residues employ auxiliary effectors, such as nonspecific nucleases, to trigger abortive infection upon detection of foreign nucleic acids. Here, we report a unique group of catalytically active pAgo proteins that frequently associate with a phospholipase D (PLD) family protein. We demonstrate that this particular system employs the catalytic center of the associated PLD protein rather than that of pAgo to restrict plasmid DNA, while interestingly, its immunity against a single‐stranded DNA virus relies on the pAgo catalytic center and is enhanced by the PLD protein. We also find that this system selectively suppresses viral DNA propagation without inducing noticeable abortive infection outcomes. Moreover, the pAgo protein alone enhances gene editing, which is unexpectedly inhibited by the PLD protein. Our data highlight the ability of catalytically active pAgo proteins to employ auxiliary proteins to strengthen the targeted eradication of different genetic invaders and underline the trend of PLD nucleases to participate in host immunity.

## Impact statement

Mesophilic prokaryotic Argonautes (pAgos) have the potential to be developed into genome editing tools. We report a mesophilic archaeal Ago that is catalytically active and utilizes an associated phospholipase D (PLD) family nuclease to restrict foreign DNA. The partnership with the PLD nuclease significantly enhances the pAgo‐based immunity against single‐stranded viruses and is essential for resisting double‐stranded plasmids. Furthermore, it shows that introducing only the pAgo protein into bacterial cells can enhance editing efficiency, suggesting its potential for genome editing applications.

## INTRODUCTION

Argonaute (Ago) proteins are widespread in all three domains of life and use small DNA or RNA guides to target complementary nucleic acids^1^, ^2^. Eukaryotic Argonautes (eAgos) are core components of RNA‐induced silencing complex (RISC) and play a key role in RNA interference (RNAi), which regulates gene expression by silencing mRNA targets in a sequence‐specific manner^3^, ^4^, ^5^. Although bacteria and archaea possess prokaryotic Argonaute proteins (pAgos), they lack RNAi pathways. Recent studies showed that some pAgos function as defense systems against invasive genetic elements, such as phages and plasmids^6^, ^7^, ^8^, ^9^.

pAgos are present in ~9% and ~32% of the sequenced bacterial and archaeal genomes, respectively. Phylogenetic analysis revealed that pAgos are much more diverse than eAgos^1^, ^10^, ^11^. Based on their domain organization and genomic context, pAgos can be separated into three groups: long‐A, long‐B, and short pAgos^11^. Similar to eAgos, both long‐A and long‐B pAgos feature a conserved core structure, including N‐terminal, L1 (linker 1), PAZ (PIWI‐Argonaute‐Zwille), L2 (linker 2), MID (middle), and PIWI (P‐element induced wimpy testis). Short pAgos consist of only two domains, MID and PIWI, but are always fused to or co‐occur in the same operon with APAZ domain‐containing protein^10^, ^11^, ^12^. The PIWI domain of long‐A pAgos contains a conserved catalytic tetrad DEDX (where X is D, H, or N) motif that chelates divalent metal ions and participates in target cleavage. Most in vitro studies show that long‐A pAgos can use small ssDNA and/or ssRNA guide oligonucleotides to cleave ssDNA and/or ssRNA targets^6^, ^13^, ^14^, ^15^, ^16^, ^17^. Long‐B pAgos lack the DEDX tetrad motif required for nuclease activity^11^, ^18^. Similarly, the DEDX catalytic tetrad is also incomplete in short pAgos. Most of the Long‐B and short pAgos co‐occur with genes that encode predicted helicases, nucleases, and other proteins^11^.

To date, many long‐A pAgos have been shown to utilize small DNA or RNA as guides to bind and cleave complementary DNA targets. The nuclease activity of pAgo was initially studied experimentally in thermophilic prokaryotes, including TtAgo (*Thermus thermophilus*)^6^, PfAgo (*Pyrococcus furiosus*)^19^, and MjAgo (*Methanocaldococcus jannaschii*)^20^. Recently, pAgos from mesophilic prokaryotes have been demonstrated to mediate DNA‐guided DNA cleavage at ambient temperatures, including LrAgo (*Limnothrix rosea*)^16^, CbAgo (*Clostridium butyricum*)^15^, ^16^, SeAgo (*Synechococcus elongatus*)^21^, KmAgo (*Kurthia massiliensis*)^22^, ^23^, IbAgo (*Intestinibacter bartlettii*)^24^, DloAgo (*Dorea longicatena*)^25^, and EmaAgo (*Exiguobacterium marinum*)^25^. In addition to guide‐dependent cleavage of targets, some pAgos, such as TtAgo^26^, MjAgo^20^, LrAgo^16^, CbAgo CbAgo^16^, SeAgo^21^, CdAgo^27^, and EmaAgo^25^, perform DNA “chopping” of double‐stranded plasmid DNA in a guide‐independent manner and exhibit nonspecific nuclease activity.

DNA‐guided cleavage of DNA targets by pAgo proteins provides the potential for the use of pAgos as a programmable tool for genome editing^2^. However, the catalytically active pAgos from thermophiles function at elevated temperatures, which limits the application of pAgos as a genome editing tool in mesophilic organisms. The mesophilic pAgos, which work at human physiological temperature, have the potential for programmable genome editing in mesophilic organisms. Owing to the lack of helicase activity, cleavage of the dsDNA target mediated by mesophilic pAgos is impeded by the melting of DNA duplex. It has been reported that the enzymatic activity of pAgos can be enhanced by combining pAgo with helicase^28^, ^29^. Interestingly, NgAgo from the halophilic archaeon *Natronobacterium gregoryi* can enhance homologous recombination and gene editing in a guide‐dependent manner^30^. Moreover, recent studies demonstrate that CbAgo can be guided by plasmid‐derived DNA guide sequences to cleave chromosome and induce homologous recombination, which suggests the potential of mesophilic pAgos to be employed for genome editing^31^, ^32^.

Notably, recent studies showed that some short pAgos, jointly with their associated proteins, functioned as a prokaryotic immune system by mediating an abortive infection (Abi) response^8^, ^9^, ^33^, ^34^. Similar to short pAgos, some long pAgos were found in association with putative nucleases from the Mrr or phospholipase D (PLD) protein families, which are anticipated or demonstrated to play a vital role in the pAgo‐mediated immunity^3^, ^10^, ^35^. The PLD superfamily proteins feature a highly conserved amino acid combination known as the “HxK” motif (“HxK(x)_4_D(x)_6_GSxN,” where x is any amino acid), which constitutes their catalytic center^36^, ^37^. PLD nucleases are ubiquitous in bacteria, plants, and mammals and are involved in various physiological processes^38^. Several well‐studied bacterial nucleases belong to the PLD superfamily, such as the IncN plasmid‐encoded endonuclease Nuc^39^ and the metal ion‐independent restriction enzyme BfiI^40^. Remarkably, a PLD domain‐containing protein was recently reported to participate in DNA phosphorothioation‐based antiviral defence^41^. It was early noticed that genes encoding the long‐A pAgo proteins are frequently associated with PLD nuclease genes in haloarchaeal genomes, but interestingly, not in other microbial genomes^10^. The exact involvement of these Ago‐associated PLD proteins (hereafter termed AgaPs) in pAgo‐based immunity remains unclear, especially considering that the associated pAgo proteins usually possess a complete DEDX catalytic tetrad.

In this study, we demonstrated that the haloarchaeal pAgo‐AgaP (APAP) system restricted plasmid DNA essentially depending on the PLD protein AgaP, but it resisted a single‐stranded DNA virus dependent on both the catalytic activity of AgaP and of pAgo. Notably, the AgaP adopts a unique split “HxK(x)_49_GSxN” motif (X denotes any amino acid), where “HxK” and “GSxN” are separated by approximately 50 amino acids. Our findings further showed that this auxiliary PLD‐associated pAgo system confers viral immunity not by inducing an Abi response but rather by actively and selectively suppressing the intracellular propagation of viral DNA, which distinguishes from the Abi‐dependent immunity recently reported for short and long‐B pAgos^35^, ^42^. At last, we employed this mesophilic pAgo system to improve gene editing in *Escherichia coli*, which was interestingly inhibited by the presence of the PLD protein.

## RESULTS

### Genetic and transcriptional link between pAgos and PLD proteins in haloarchaea

**Figure 1:**
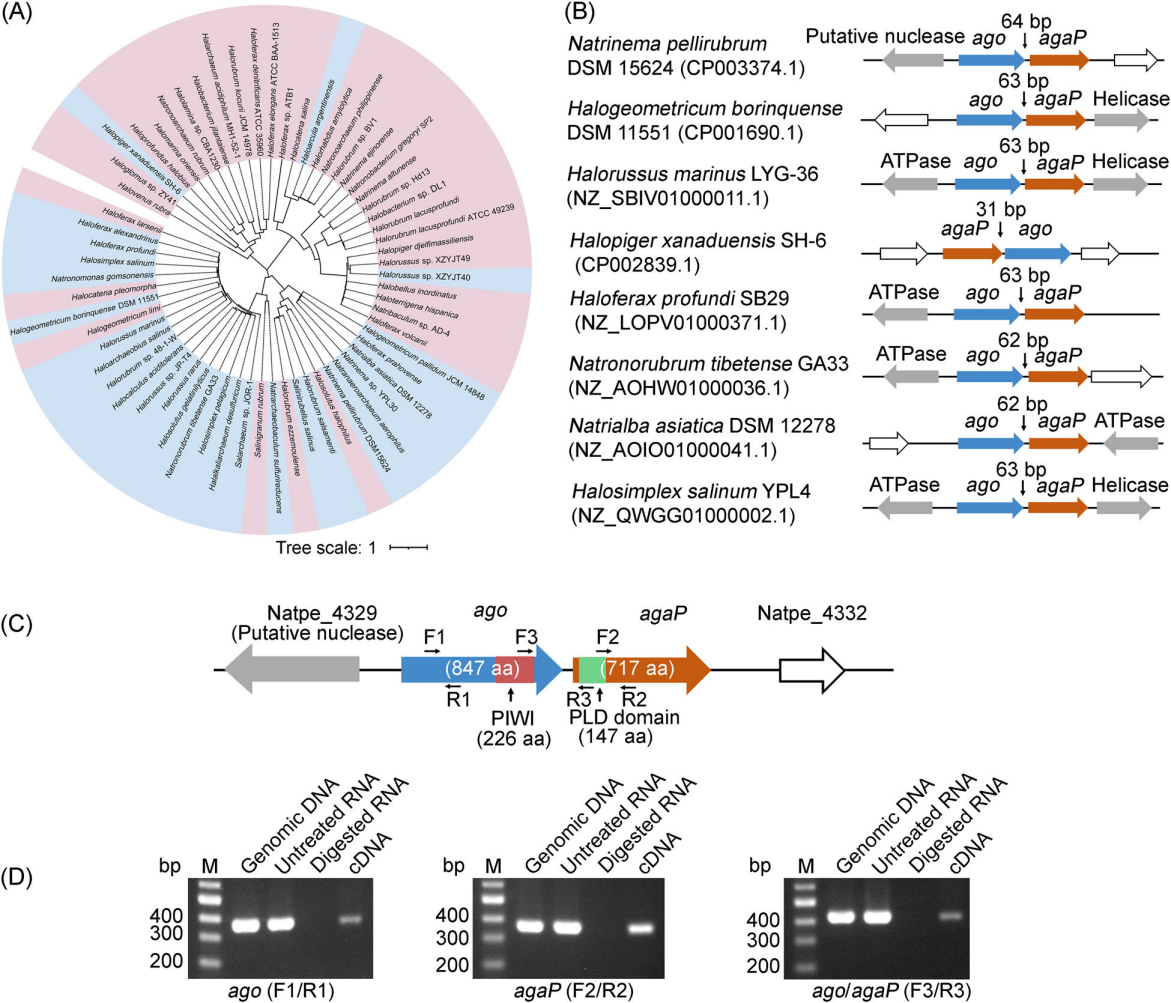
Haloarchaeal pAgo proteins associate with a phospholipase D (PLD) family protein. (A) Maximum likelihood phylogeny of all identified pAgos in available haloarchaeal genomes. The pAgos that associate with an AgaP are shaded in blue. (B) Representative *ago‐agaP* operons, with the size (bp, base pair) of each intergenic region indicated. (C) The *ago‐agaP* operon in *Natrinema pellirubrum* DSM 15624, with genes encoding a hypothetical protein shown in white. The primers used for reverse‐transcription‐PCR (RT‐PCR) are indicated. (D) Profiling the transcription pattern of the *ago*‐*agaP* operon using RT‐PCR. Genomic DNA, untreated total RNA (with DNA contaminants), and DNA‐digested total RNA from *N. pellirubrum* cells were separately used as templates for control. cDNA was generated by reverse transcription of the DNA‐free RNA sample using random primers (see Materials and Methods). The primers used for each assay are indicated below the corresponding gel. M, dsDNA size marker.

Early bioinformatic analyses revealed that, in haloarchaeal genomes, the *ago* genes are frequently accompanied by genes encoding proteins with an N‐terminal PLD family domain^10^, which we designated *agaP* (encoding AgaP) genes. Interestingly, multiple sequence alignment analysis of the haloarchaeal pAgo proteins showed that their PIWI domain consistently contains the conserved catalytic tetrad DEDX motif (Figure S1), which suggests that they per se likely possess the nuclease activity and leads to the question that whether and how the PLD proteins are involved in the immunity mediated by haloarchaeal pAgos. To fully understand the evolutionary link between *ago* and *agaP* genes in haloarachaea, we systemically investigated the available haloarchaeal genomes in NCBI and found that ~9.6% (63 out of 656) of haloarchaeal species have *ago* genes, of which ~44.4% (28 out of 63) are associated with AgaPs (Figure 1A). In Figure 1B, we showed the gene organization of some representative examples. Apparently, the long‐active pAgo proteins from haloarchaea are tightly associated with the PLD family proteins during evolution. We named pAgo‐PLD systems “APAP” (long‐A prokaryotic Argonaute PLD) systems based on the nomenclature proposed for other pAgo systems^34^, ^35^.

The *agaP* genes can locate upstream or downstream of their associated *ago* genes, usually with a short intergenic region ranging from 31 to 64 bp (Figure 1B), suggesting they should be organized into a single operon. Then we characterized the transcription pattern of the *ago*‐*agaP* operon from *Natrinema pellirubrum* (Figure 1C), which was available in our lab. With gene‐specific primer pairs, we showed that these two genes were both actively transcribed. Using two primers, each specified as *ago* and *agaP*, respectively, we further showed that they were co‐transcribed (Figure 1D), which strongly indicates their functional coupling.

### AgaP alone causes cellular toxicity that is suppressed by pAgo

**Figure 2:**
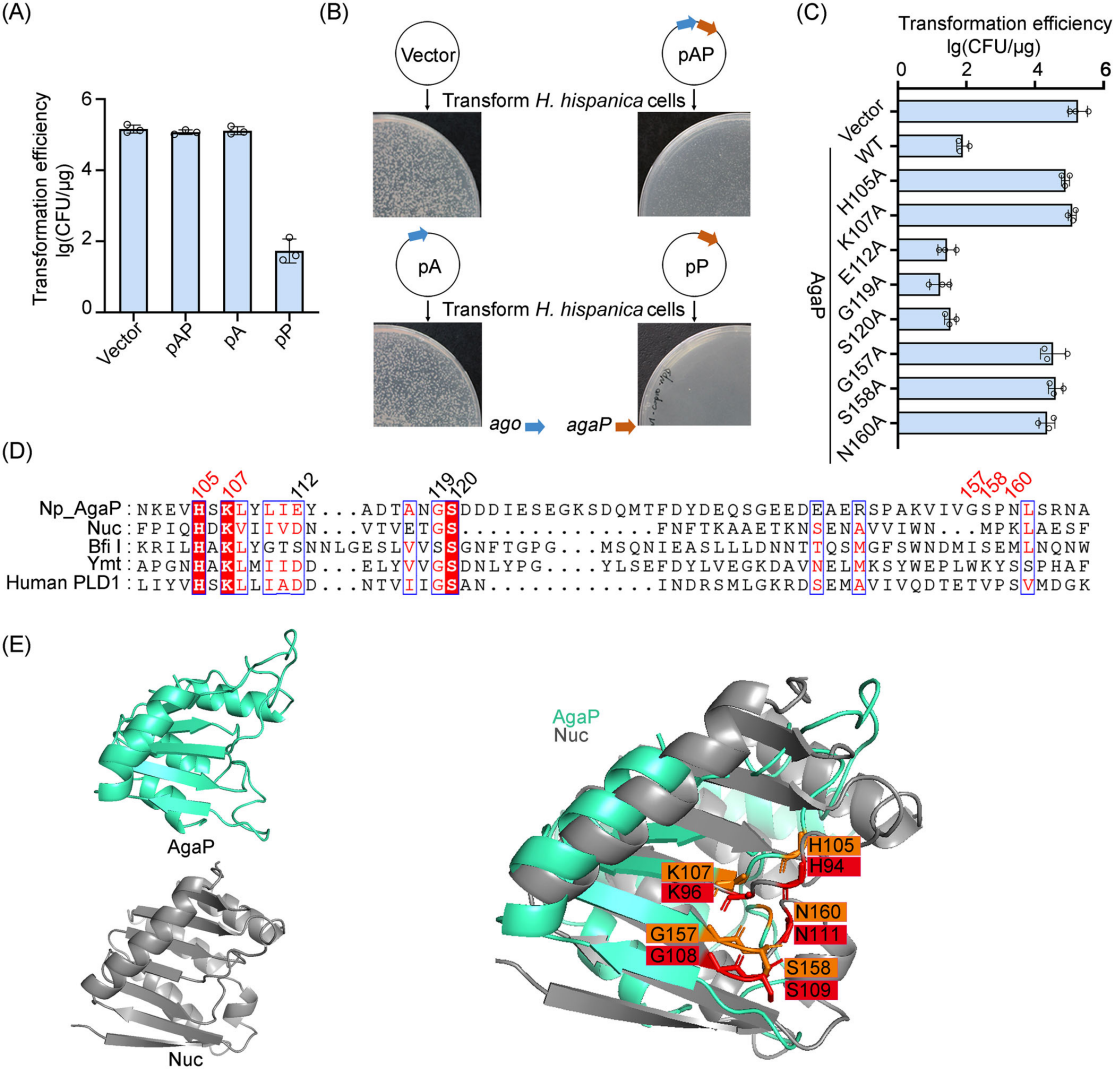
AgaP exhibits toxicity in the absence of its associated pAgo and features a catalytic motif. (A) Transformation of *Haloarcula hispanica* cells with a plasmid carrying *ago* (pA), *agaP* (pP), or both genes (pAP). (B) The resulting colonies formed by *H. hispanica* transformants on a selective medium. (C) Mutational analysis performed on AgaP. Vector, the empty pWL502. WT denotes a wild‐type AgaP. The mutated residues are indicated in panel D. Data are presented as mean value ± SD (*n* = 3). (D) Multiple sequence alignment using *N. pellirubrum* AgaP (Np_AgaP) and four well‐characterized PLD proteins. The numbers indicate the position of the residues subjected to mutation analysis, with the critical catalytic residues highlighted in red. (E) Prediction of the structure of AgaP and its comparison to Nuc. The structure of Agap was predicted using Nuc (PDB: 1BYR) as a model. The predicted catalytic residues of AgaP (highlighted in orange) align with those of Nuc (in red). The images were visualized using PyMOL. CFU, colony‐forming unit.

Since genetic manipulation systems have not been developed for *N. pellirubrum*, we dissected its *ago*‐*agaP* operon in the well‐studied model haloarchaeon, *Haloarcula hispanica* ATCC 33960. Because the *H. hispanica* genome encodes a type I‐B CRISPR‐Cas system that can actively defend the host cell against a target plasmid or virus^43^, ^44^, we used the *cas6*‐deleted strain (designated WT) in this study to facilitate assessing the immune effects of the heterologous pAgo system. We first cloned the complete *ago*‐*agaP* operon of *N. pellirubrum* (including a 300 bp upstream sequence that may contain their promoter) into an expression vector (pWL502) and introduced it into *H. hispanica* cells by transformation. Reverse transcription (RT) assay confirmed that these two plasmid‐carried genes were actively (co‐)transcribed in this heterologous host (Figure S2). Then we engineered another two plasmids containing only *ago* or *agaP* (under the control of their shared promoter), respectively. For the plasmid carrying only *ago* or the one carrying the entire operon, we observed a high efficiency that is comparable to the empty vector (∼10^5^ CFU/μg plasmid DNA; CFU, colony‐forming unit) when transforming *H. hispanica* cells, but unexpectedly, a very low efficiency (∼10^2^ CFU/μg) was observed for the plasmid only expressing *agaP* (Figure 2A,B). We inferred that the PLD protein turned out to be toxic in the absence of the Ago protein.

### AgaP has a unique catalytic motif

The catalytic center of nearly all PLD superfamily members is formed by a highly conserved amino acid sequence, "HxK(x)_4_D(x)_6_GSxN" (where x is any amino acid), termed the "HxK" motif^36^, ^37^. By multiple sequence alignment with four well‐characterized PLD family proteins, we showed that the *N. pellirubrum* AgaP also possesses this conserved motif, except that the aspartic acid (D) in the “HxK(x)_4_D(x)_6_GSxN” sequence is replaced by the other acidic amino acid glutamic acid (Glu112), and the amidic asparagine (N) replaced by the acidic aspartic acid (Asp122) (Figure 2D). Then, we predicted the structure of AgaP using Phyre2 and aligned it to the reported crystal structure of Nuc (an endonuclease of the PLD family from *Salmonella typhimurium*, PDB: 1BYR). His105 and Lys107 of AgaP perfectly align with the corresponding core residues His94 and Lys96 of Nuc. However, interestingly, instead of the residues Gly119, Ser120, and Asp122 within the predicted catalytic motif, three remote amino acids, Gly157, Ser158, and Asn160, perfectly aligned to the corresponding catalytic residues of Nuc and clustered with His105 and Lys107 to form the catalytic center (Figure 2E).

Then, we engineered plasmids to express a series of AgaP derivates, each with a specific residue mutated to alanine. Subsequently, we tested their cellular toxicity (in the absence of NpAgo) in *H. hispanica* cells. Interestingly, the core residue mutants H105A and K107A exhibited a complete loss of toxicity, and their corresponding plasmids demonstrated a transformation efficiency that was almost equivalent to the empty vector (∼10^5^ CFU/μg) (Figure 2C). It was indicated that the catalytic activity of AgaP plays a critical role in causing cellular toxicity. By contrast, E112A, G119A, and S120A retained the toxicity and resulted in a very low transformation efficiency (∼10^2^ CFU/μg), akin to the plasmid expressing the wild‐type (wt) AgaP. Instead, mutating the residues Gly157, Ser158, and Asn160, which are remote from the critical “HxK” motif but predicted to participate in forming the catalytic center by structure modeling, subverted the toxicity and resulted in a high transformation efficiency (equivalent to the empty vector) (Figure 2C). By aligning the *N. pellirubrum* AgaP to its most related homologs, we further showed that this remote “GSxN” motif is very conserved, while little conservation was observed for Gly119, Ser120, or Asp122 (Figure S3). Therefore, we conclude that the AgaPs feature a catalytic motif that is separated by ~50 amino acids (between the core “HxK” residues and the “GSxN” residues) in primary sequence but retain the ability to form the catalytic center in tertiary structure.

### APAP system restricts plasmid DNA

**Figure 3:**
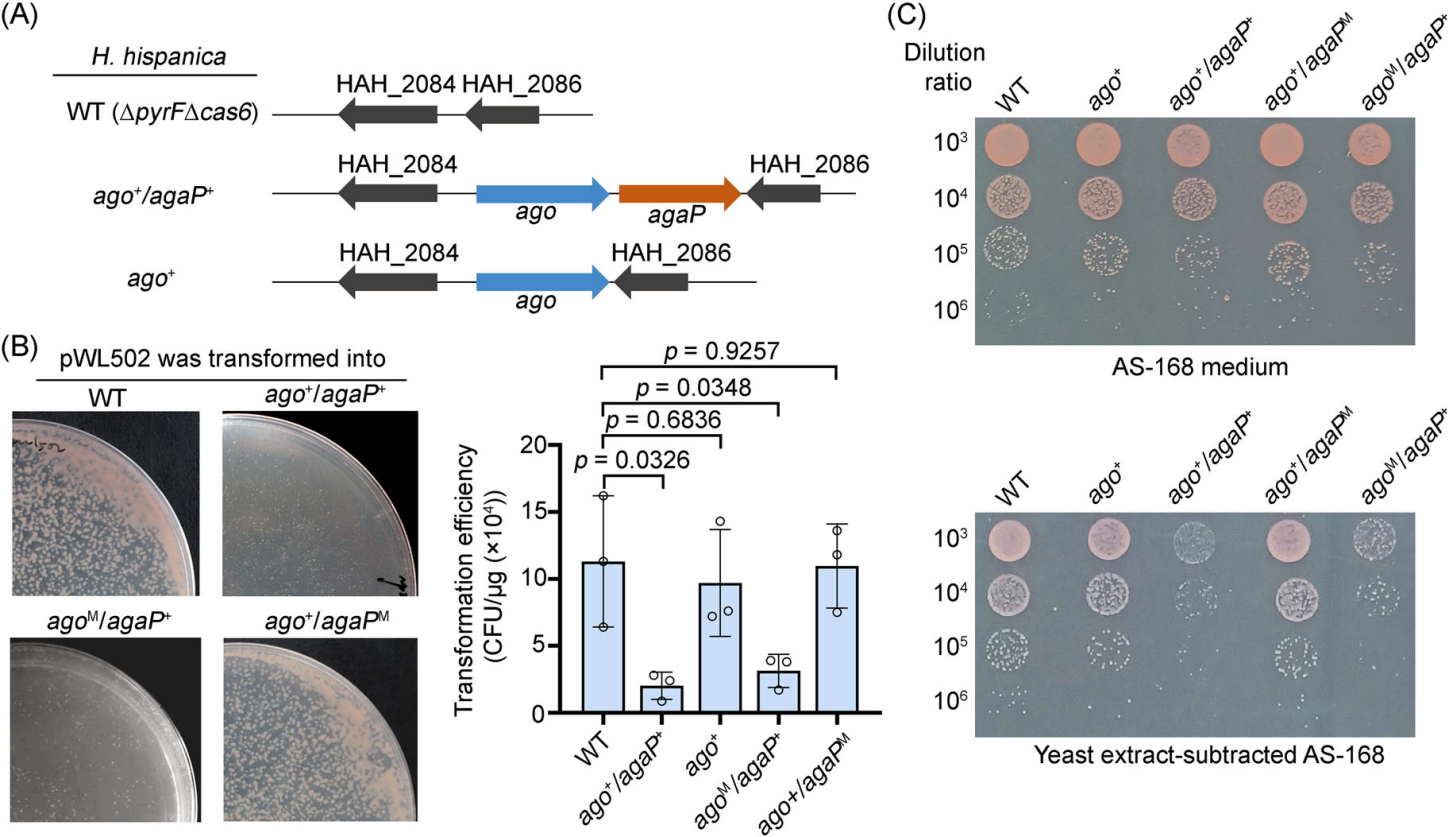
AgaP is indispensable for the immunity against plasmid DNA. (A) Schematic representation of the *H. hispanica* mutants. The *ago* gene or the *ago‐agaP* operon from *N. pellirubrum* was integrated into *H. hispanica* chromosome at the genomic location of *pyrF* (HAH_2085), which had been deleted to create an auxotrophic *H. hispanica* strain. (B) Transformation efficiency of *H. hispanica* cells by pWL502. The photographs display the colonies formed on selective plates. Data are presented as mean value ± SD (*n* = 3). The *p* values were obtained from a two‐sided *t*‐test. (C) Comparison of cell growth between the WT strain and mutant strains on plates. The transformants were diluted and plated on nonselective (AS‐168) and selective (yeast extract‐subtracted AS‐168) medium, respectively. The *ago* ^+^/*agaP* ^M^ mutant encodes a catalytically dead AgaP, with both H105 and K107 mutated to alanine. The ago^M^/agaP^+^ variant encodes a catalytic mutant Ago, with D630 mutated to alanine.

During plasmid expression of the *N. pellirubrum* Ago (NpAgo) and AgaP, we noticed that the *H. hispanica* cells co‐expressing both proteins formed much smaller colonies on the selective medium, compared to cells expressing only NpAgo or carrying the empty vector (Figure 2B). This implies that the co‐expression of NpAgo and AgaP may cause a moderate level of cellular toxicity or that these two proteins together produce immunity against the plasmid expressing them. Starting from the WT, we integrated the entire *N. pellirubrum ago‐agaP* operon (along with its native promoter) into the *H. hispanica* chromosome, resulting in the knock‐in mutant (designated *ago* ^+^ */agaP* ^+^) (Figure 3A). By this means, we also constructed the *ago* ^+^ mutant where only the *N. pellirubrum ago* gene was inserted into the chromosome but failed to construct the *agaP^+^* mutant, which reaffirmed its toxicity in the absence of the pAgo partner (Figure 2A,B). When transforming these mutants with empty pWL502, the transformation efficiency of *ago* ^+^ */agaP^+^* cells was reduced by ~82% compared to that of WT (*p* = 0.0326), while no significant difference was observed between WT and *ago^+^* cells (*p* = 0.6836) (Figure 3B). Moreover, the *ago* ^+^ */agaP* ^+^ cells formed colonies that were noticeably smaller than those of WT (Figure 3B). Therefore, the co‐expression of *N. pellirubrum* Ago and AgaP together in *H. hispanica* cells restricts plasmid transformation.

We inoculated these colonies into a liquid medium lacking yeast extract (to maintain the *pyrF*‐bearing plasmid) and cultivated them until they reached a similar cell concentration (by monitoring the optical density). Following serial dilution, we separately spotted these cell cultures onto selective and nonselective plates. On the nonselective plates (containing yeast extract), the WT, *ago* ^+^, and *ago* ^+^ */agaP* ^+^ cells formed a nearly equivalent number of colonies at the same dilution gradient (e.g., 10^6^) (Figure 3C), which confirmed that these cultures were harvested with similar cell densities. However, on the selective plates lacking yeast extract, the colonies of *ago* ^+^ */agaP* ^+^ were reduced by one order of magnitude compared to the WT or *ago^+^* colonies (Figure 3C), indicating nearly 90% of *ago* ^+^ */agaP* ^+^ individuals had lost the plasmid. Therefore, the pAgo system not only limited plasmid transformation but also effectively expelled the intracellular plasmid DNA under nonselective conditions. In addition, when we mutated the catalytic motif of AgaP, the resulting *ago* ^+^/*agaP* ^M^ cells showed a plasmid‐transforming efficiency comparable to the WT cells (*p* = 0.9257; Figure 3B), and expulsion of intracellular plasmid DNA was no longer observed (Figure 3C). Therefore, the catalytic activity of AgaP is essential for the observed plasmid resistance.

Typically, pAgo proteins that associate with additional effectors are either long‐B or short pAgos, both of which lack the DEDX catalytic center. This leads us to question whether the catalytic center of NpAgo is required for the observed plasmid immunity. Notably, when the conserved catalytic residue D630 of NpAgo was mutated (resulting in *ago* ^M^ */agaP* ^+^), a ~74% reduction in transformation efficiency was still observed compared to the WT strain (*p* = 0.0348; Figure 3B). In addition, similar to *ago* ^+^ */agaP* ^+^ cells, *ago* ^M^ */agaP* ^+^ cells transformed by pWL502 formed colonies that were noticeably smaller than transformed WT cells (Figure 3B). During the dilution plating assay, a 1‐log reduction in colony number on selective medium (compared to the number on nonselective medium) was observed for the *ago* ^M^ */agaP* ^+^ transformants (Figure 3C), indicating nearly 90% *ago* ^M^ */agaP* ^+^ cells quickly lost the plasmid in the absence of selection. These data demonstrate that the catalytic activity of NpAgo is dispensable for plasmid resistance. In conclusion, NpAgo relies on the catalytic activity of associated AgaP, rather than its own DEDX catalytic center, to achieve immunity against plasmid DNA. We also attempted to dissect the role of the MID domain (anchors the 5′‐end of guide) in plasmid immunity, but construction of a strain encoding both a MID‐mutated NpAgo (Y570A/K574 A) and AgaP was unsuccessful, suggesting the MID domain is possibly important for inhibiting the toxicity of AgaP.

### Virus immunity relies on the catalytic center of NpAgo and is enhanced by AgaP

**Figure 4:**
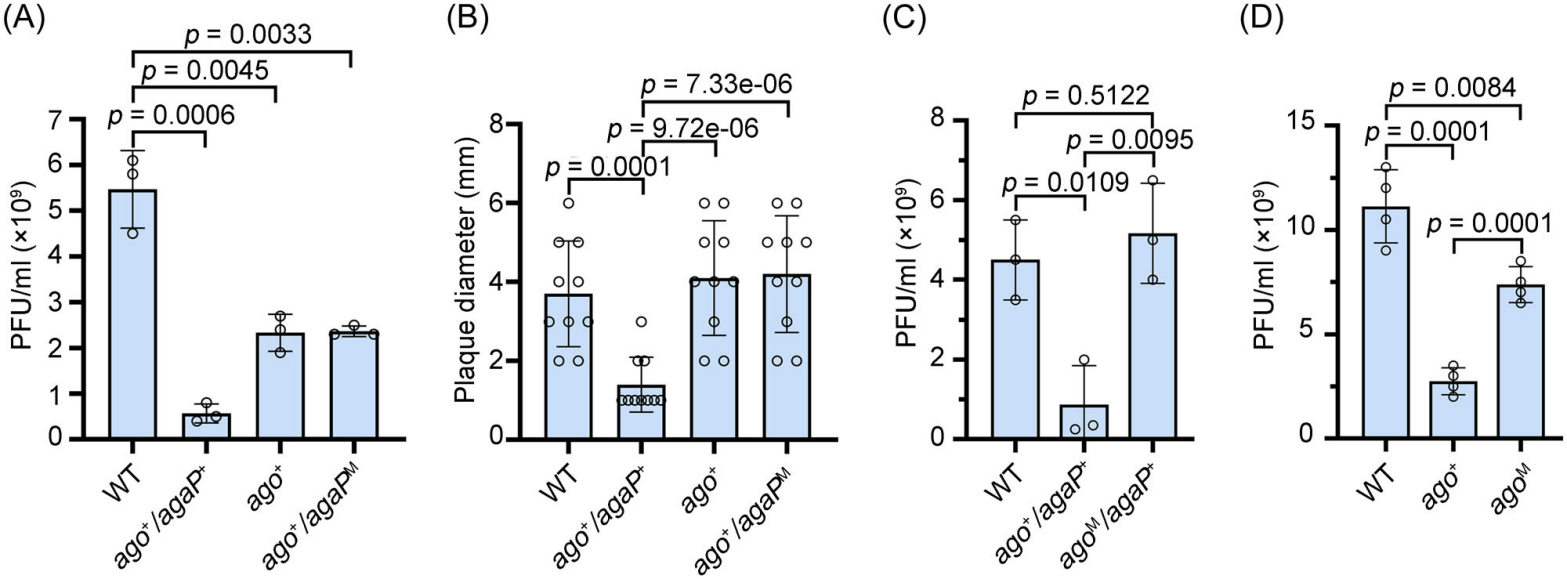
AgaP enhances the pAgo‐based immunity against virus. (A) Plaque forming unit (PFU) of the same HHPV‐2 dilution on lawns of *H. hispanica* WT, *ago* ^+^, *ago* ^+^/*agaP* ^+^, or *ago* ^+^/*agaP* ^M^ cells. Data are presented as mean value ± SD (*n* = 3). (B) The size of HHPV‐2 plaques formed on different *H. hispanica* cells. *agaP* ^M^ encodes a catalytically dead AgaP mutant (H105A/K107A). (C) PFU of the same HHPV‐2 dilution on lawns of *H. hispanica* WT, *ago* ^+^/*agaP* ^+^, or *ago* ^M^/*agaP* ^+^ cells. The HHPV‐2 used in panel A and C were prepared from different batches. Data are presented as mean value ± SD (*n* = 3). (D) PFU of the same HHPV‐2 dilution on lawns of WT *H. hispanica* or a derivate encoding a wild‐type *N. pellirubrum* pAgo (*ago* ^+^) or its catalytically dead mutant *ago* ^M^ (D630A). Data are presented as mean value ± SD (*n* = 4). *p* values were obtained from a two‐sided *t*‐test. PFU, plaque‐forming unit.

The resistance to plasmid DNA suggests that the APAP system may also play a protective role against virus infections. Therefore, we infected these knock‐in mutants with *H. hispanica* pleomorphic virus‐2 (HHPV‐2), a circular single‐stranded DNA halovirus^43^. Remarkably, the *ago* ^+^ */agaP* ^+^ mutant exhibited a significant reduction (approximately 90%) in plaque‐forming units (PFUs) compared to the WT strain (*p* = 0.0006), demonstrating the virus immunity conferred by this system (Figure 4A). Intriguingly, a smaller reduction in PFU (approximately 57%) was also observed when the AgaP protein was absent (*ago* ^+^) or when its catalytic motif was mutated (*ago* ^+^/*agaP* ^M^) (*p* = 0.0045 or 0.0033). This suggests that the expression of only the pAgo protein provides a lower level of virus immunity. Furthermore, upon closer examination of the plaques, we noticed that the plaques formed on the lawn of *ago* ^+^/*agaP^+^* cells were significantly smaller than those formed on the lawn of WT, *ago* ^+^, or *ago* ^+^/*agaP* ^M^ cells (*p* = 0.0001, *p* = 9.76e−06, or *p* = 7.33e−06; Figure 4B). Therefore, we conclude that the PLD protein and its catalytic activity are important, although maybe not essential, for the observed immunity against HHPV‐2.

Then we tested the necessity of the catalytic center of NpAgo in this antiviral process. Upon evaluating the virus susceptibility of *ago* ^M^ */agaP* ^+^ cells, we observed a high level of PFU that was equivalent to that observed for WT cells (Figure 4C). These data demonstrated the critical role of the catalytic center of NpAgo during the immunity against HHPV‐2. However, interestingly, when we compared the virus susceptibility of *ago* ^+^ and *ago* ^M^ cells, the active NpAgo resulted in a substantial decrease (~75%) in PFU compared to the WT strain, while the dead NpAgo caused a smaller reduction in PFU (~33%) (Figure 4D). Therefore, in the absence of AgaP, a catalytically inactive NpAgo can still confer *H. hispanica* cells with a certain level of protection against HHPV‐2.

### Ago/AgaP actively suppresses the propagation of viral DNA

**Figure 5:**
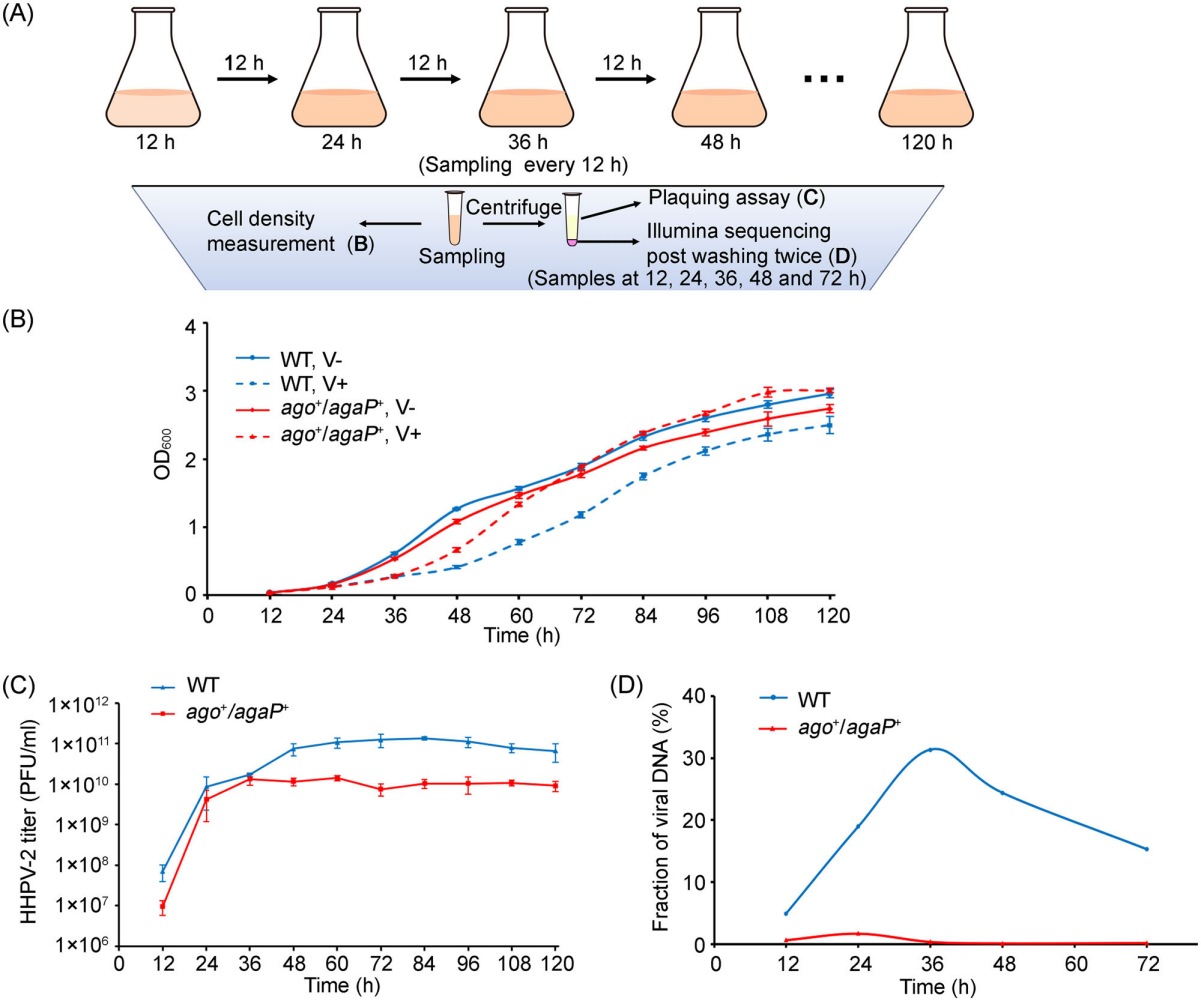
The NpAgo system effectively suppresses HHPV‐2 propagation in *H. hispanica* cells. (A) Schematic illustration of the experiment procedure. Cultures were serially sampled every 12 h to monitor cell density (panel B), after which the samples were centrifuged, and the supernatants were collected for plaque assays to determine the titer of free virus particles (panel C). The precipitated cells collected at indicated time points were subjected to total DNA extraction and Illumina sequencing to determine the ratio of intracellular viral DNA (panel D). (B) The growth curve of WT or *ago* ^+^/*agaP* ^+^ *H. hispanica* cells infected (V+) or uninfected (V−) by HHPV‐2. (C) The titer of free virus particles released from *H. hispanica* cells during the growth curve. Data are presented as mean value ± SD (*n* = 3). (D) The ratio of intracellular viral DNA to total DNA in infected WT or *ago* ^+^/*agaP* ^+^ cells.

The immune strategy that involves the suicidal of virus‐infected cells to protect the uninfected cells, termed Abi, is employed by several pAgos to provide antiviral defense on the population level^8^, ^9^, ^33^, ^34^, ^35^. The cellular toxicity of AgaP in the absence of its pAgo partner raised the possibility that this system may confer virus immunity by inducing an Abi response. To investigate this possibility, we infected *H. hispanica* strains with HHPV‐2 at a high multiplicity of infection (MOI) of 10 to ensure that every individual cell in the culture was infected by at least one viral particle. Cultures were serially collected at different time points, and the samples were subjected to optical density measurement, plaque assay, or Illumina sequencing (Figure 5A). Without virus infection, the *ago* ^+^/*agaP* ^+^ strain displayed a growth curve similar to WT (Figure 5B), indicating the expression of this immune system does not cause marked advantages or downsides on cell growth. Notably, HHPV‐2 infection caused a noticeable retardation in the growth curve of both WT and *ago* ^+^/*agaP* ^+^ strains. However, after 36 hours post infection (hpi), the growth curve of *ago* ^+^/*agaP* ^+^ started to recover more quickly than that of WT (Figure 5B). To confirm this result, we repeated the assay three times and observed similar trends. These findings suggest that in *H. hispanica* cells, the *N. pellirubrum* APAP system starts to produce active immunity against HHPV‐2 before 36 hpi without inducing cell death/dormancy. Therefore, we conclude that this auxiliary PLD‐associated pAgo system does not employ the Abi strategy.

Along the growth curve, we consecutively collected samples from the infected WT and *ago* ^+^/*agaP* ^+^ cultures and separated the cells from the culture using centrifugation. We first determined the titer of free virus particles in each supernatant using the plaquing assay. Interestingly, both the WT and *ago* ^+^/*agaP* ^+^ cultures showed a similar increase in virus titer before 36 hpi. However, beyond this time point, the viral particles released from WT cells further increased by approximately 10‐fold, while no significant increase was observed for those from *ago* ^+^/*agaP* ^+^ cells (Figure 5C). Subsequently, we analyzed the intracellular viral DNA content by subjecting the separated cells to Illumina sequencing after washing them twice with a fresh medium. Remarkably, the ratio of viral DNA in WT cells rapidly increased, reaching 31.35% of the total DNA at 36 hpi, and then started to decline (Figure 5D). This finding is consistent with the observed release of massive virus progenies from WT cells after 36 hpi (Figure 5C). In contrast, the ratio of HHPV‐2 DNA in *ago* ^+^/*agaP* ^+^ cells never exceeded 1.7% throughout the entire infection assay and descended to ~0.1% at 48 and 72 hpi (Figure 5D). These data demonstrate that the APAP system effectively suppresses the propagation of viral DNA in *H. hispanica* cells and, as a result, significantly reduces the production of viral progenies.

### NpAgo alone enhances gene editing in *E. coli*

**Figure 6:**
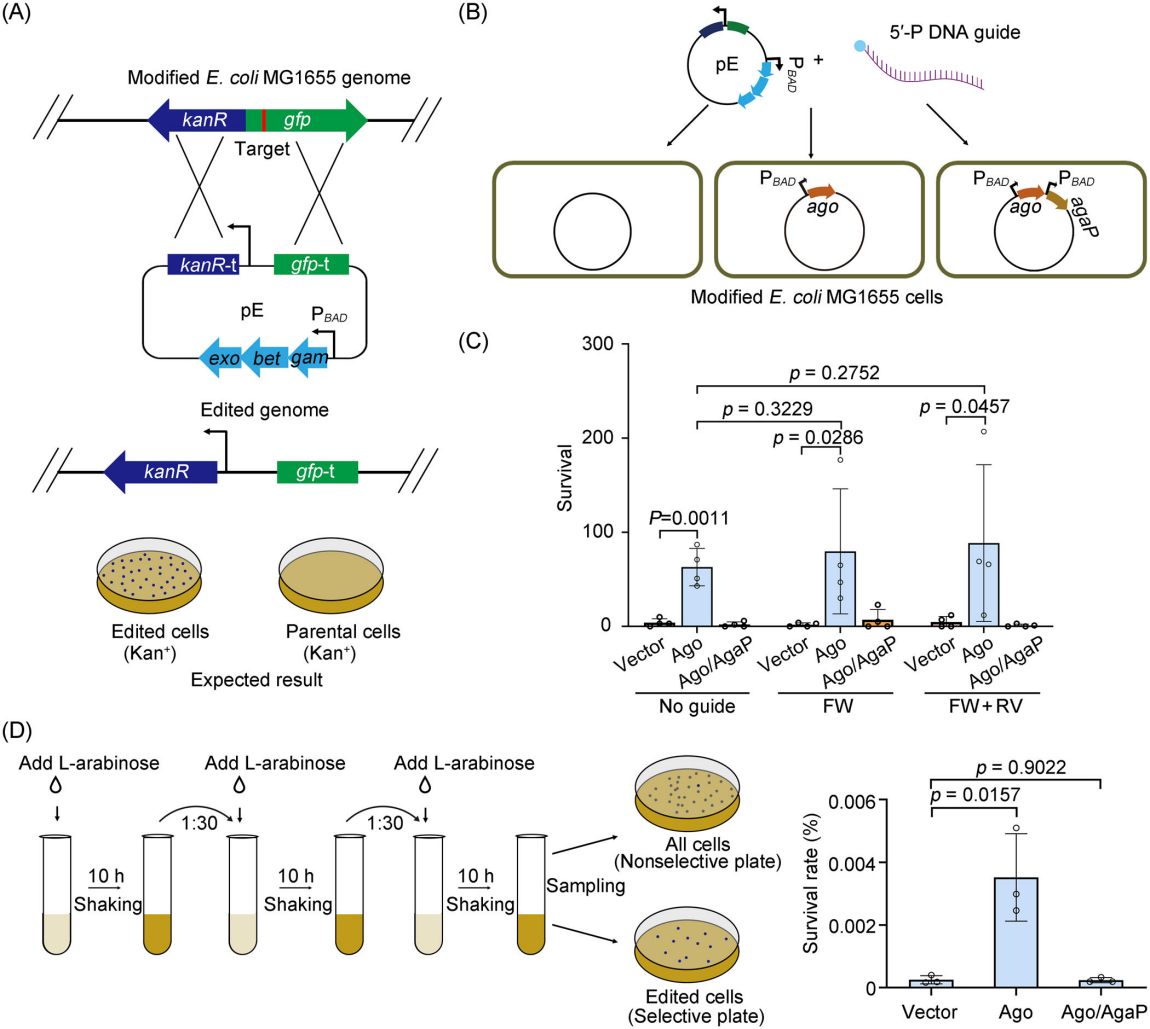
NpAgo alone enhances gene editing in *Escherichia coli*. (A) Schematic representation of the experimental design for testing NpAgo‐assisted gene editing in *E. coli*. *E. coli* MG1655 was modified by inserting a promoter‐lacking *kanR* gene and a *gfp* gene into chromosome. The editing plasmid pE contains a truncated *kanR* (*kanR*‐t) with a constitutive promoter, the λ‐Red system, under the control of an l‐arabinose‐inducible P_*BAD*_ promoter, and homology arms. Recombination between chromosomal DNA and the editing plasmid results in edited cells that are able to grow and form colonies on selective media due to the introduction of the constitutive promoter to drive the transcription of *kanR*. The target site is indicated by a red line. (B) Transformation of modified *E. coli* MG1655 cells with editing plasmid and DNA guides. *E. coli* cells expressing only NpAgo or both NpAgo and AgaP were transformed with pE and DNA guides. The expression of Ago (and AgaP) was induced by l‐arabinose. (C) The number of *E. coli* cells transformed by pE with or without DNA guides and survived on a kanamycin‐containing medium. FW, forward guide; RV, reverse guide. Data are presented as mean value ± SD (*n* = 4). (D) Evaluation of the effect of NpAgo on homologous recombination. *E. coli* cells transformed with pE and the *ago*‐expressing plasmid serially cultivated. l‐Arabinose was added to the medium to induce the expression of λ‐Red and NpAgo (or NpAgo and AgaP). Cultures were plated onto selective or nonselective media after serial dilution, and the ratios of survivors on selective plates to those on nonselective plates were calculated and plotted based on three independent biological replicates. Data are presented as mean value ± SD (*n* = 3). *p* values were obtained from two‐tailed Student's *t*‐tests.

The plasmid restriction capability of the *N. pellirubrum* APAP system at ambient temperatures implies its potential for development as a gene editing tool in mesophilic organisms. We tested this potential in *E. coli* cells (Figure 6). According to a previous study^30^, we first engineered a derivate of *E. coli* MG1655, which carried a promoter‐lacking kanamycin resistance (*kanR*) gene and a green fluorescence (*gfp*) gene on its chromosome (Figure 6A). A 24‐bp sequence at the start of the *gfp* gene was selected as the target for guide DNA design. Although the guide molecule of NpAgo has not been characterized, we inferred that this Ago protein may use 5′‐phosphorylated oligonucleotides as guides based on the recent literature about related long‐A pAgo proteins^16^, ^19^, ^20^, ^22^, ^23^, ^30^. We then constructed an editing plasmid pE to provide the λ‐Red system and a donor DNA, which consists of two homology arms (one being the first ~500 bp of *knaR*, and the other being a ~500 bp sequence downstream of the target site within *gfp*) for recombination and an intervening sequence containing a constitutive promoter (Figure 6A). In theory, parental cells would not be able to survive the selection pressure of kanamycin, while the recombinants could survive due to the addition of the promoter to *kanR* via homologous recombination between the editing plasmid and the chromosome (Figure 6A).

After codon optimization for expression in *E. coli*, we introduced a plasmid expressing only NpAgo or expressing both NpAgo and AgaP into the modified *E. coli* MG1655 cells and then transformed these cells by the editing plasmid pE, with or without 5′‐phosphorylated ssDNA guides (Figure 6B). Interestingly, regardless of the presence of a single DNA guide or a pair of guides complementary to the opposite DNA strands, the number of survivors on selective medium (Kan^+^) markedly increased when only NpAgo was expressed but not when both NpAgo and AgaP were expressed (Figure 6C). This finding indicates that NpAgo enhances the editing efficiency of the *E. coli* genome independent of additive DNA guides, which is inhibited by its interaction with AgaP.

To further verify the feasibility of NpAgo used for improving editing efficiency, we continuously cultivated the *E. coli* cells co‐transformed with the NpAgo‐expressing plasmid (or the plasmid expressing both NpAgo and AgaP) and the editing plasmid in medium containing l‐arabinose (inducing the expression of λ‐Red and Ago (or Ago‐AgaP)). The cells were cultured for three passages to promote recombination, and then the cultures from the final passage were serially diluted and plated on the selective (only edited individuals can grow) or nonselective (all cells can grow) solid medium (Figure 6D). As expected, the percentage of edited individuals increased by approximately 14‐fold with the expression of only NpAgo (*p* = 0.0157). Importantly, this effect was not observed when AgaP was simultaneously provided, consistent with the data in Figure 6C. We randomly selected 10 edited individuals from the NpAgo‐expressing group, and by DNA sequencing, we validated the precise insertion of the designed promoter sequences into their genome (Figure S4). Subsequent PCR analysis showed that the editing plasmid was also retained in the kanamycin‐resistant cells after recombination (Figure S4D). Additionally, we used the editing plasmid pE2, which only contains a donor DNA for recombination, and the NpAgo‐expressing plasmid to test the survival rate in the absence of the λ‐Red system according to the procedure shown in Figure 6D. We found that the percentage of edited individuals was not increased when NpAgo was expressed (Figure S5). Therefore, we conclude that the haloarchaeal protein NpAgo can be utilized to enhance gene editing efficiency in the presence of λ‐Red system in *E. coli*, which can be inhibited by AgaP.

## DISCUSSION

Recent studies have uncovered that pAgo proteins play a role in defending against mobile genetic elements (MGEs)^6^, ^7^, ^8^, ^9^. Similar to eukaryotic Ago proteins, the long‐A pAgos possess a conserved catalytic tetrad DEDX, allowing them to directly target MGEs or MGE‐encoded transcripts. In contrast, long‐B and short pAgos usually lack an intact DEDX tetrad, and notably, these catalytically inactive pAgos were recently reported to utilize a variety of auxiliary effectors, including nucleases, NADases, and transmembrane proteins, to trigger Abi upon detection of target MGEs^42^. Approximately 10% of haloarchaeal genomes encode long‐A pAgo proteins, and interestingly, almost half of the haloarchaeal *ago* genes are associated with either a downstream or upstream gene encoding a PLD family protein (Figure 1), termed AgaP (Ago‐associated PLD protein) in this study. The exact role of this auxiliary protein in the immunity process mediated by these catalytically active pAgo proteins remains unclear.

In this study, we demonstrated that the pAgo protein from *N. pellirubrum* and its associated AgaP worked together to confer immunity against a plasmid, which resulted in a decrease in the transformation efficiency, smaller transformed colonies on selective plates, and the rapid expulsion of the plasmid in the absence of selective pressure (Figure 3). Surprisingly, the AgaP protein and its catalytic residues are essential for the observed plasmid resistance, whereas the canonical catalytic tetrad of Ago is not required. Additionally, we found that *N. pellirubrum* APAP can provide immunity against HHPV‐2, a single‐stranded DNA halovirus. This viral immunity mainly relies on the catalytic activity of pAgo, rather than that of AgaP, although AgaP does enhance the level of immunity (Figure 4C). The reliance on different catalytic components for plasmid resistance and viral immunity is likely due to the distinct DNA forms of invaders. Interestingly, a helicase‐encoding gene occurs in the neighbor of some *ago‐agaP* operons (see Figure 1B for examples), implying that ancillary helicases may be able to improve the immune effect of the APAP system.

Of note, the *N. pellirubrum* APAP system effectively suppressed the propagation of HHPV‐2 DNA in *H. hispanica* cells (Figure 5), and it seemed to be also capable of selectively eliminating the plasmid DNA (Figure 3C), indicating that this PLD‐associated pAgo system can well distinguish extrachromosomal DNA from genomic DNA. Further investigation is required to ascertain whether this discrimination depends on the high copy number of invaders or a specific genetic element. Characterizing the chemical identity and sequence preference of its oligonucleotide guides may provide valuable insights. However, our attempts to obtain soluble and stable NpAgo and AgaP proteins in *E. coli* were unsuccessful due to their salt adaptations^45^, ^46^. We also attempted to express the *N. pellirubrum* APAP system in *E. coli* but did not observe immune effects against any plasmids or phages (data not shown). Overall, it seems that haloarchaeal pAgo proteins frequently utilize PLD family proteins to actively eliminate extrachromosomal DNA, in contrast to short and long‐B pAgos, which employ auxiliary nucleases (or other effectors) to trigger an Abi response upon detection of foreign DNA^8^, ^9^, ^33^, ^34^, ^35^. However, interestingly, introducing only AgaP into *H. hispanica* cells resulted in very few viable cells (Figure 2), similar to what has been observed for the effector proteins associated with inactive pAgos, such as the TIR‐APAZ protein of SPARTA (short prokaryotic Argonaute TIR‐APAZ) systems and the bAgaS protein of BPAS (long‐B prokaryotic Argonaute Sir2) systems^34^, ^35^.

Our study also revealed a distinctive arrangement of catalytic residues in the PLD proteins associating with pAgo proteins: “HxK” and “GSxN” within the core motif are separated by approximately 50 amino acids in the primary sequence. This arrangement is infrequently observed in other PLD proteins. Our data showed that AgaP alone can cause cytotoxicity, which relies on its catalytic residues. We speculate that AgaP may act as a nonspecific nuclease, and interaction with pAgo likely inhibits this activity. It is noteworthy that our attempts to create a strain expressing both AgaP and a MID‐mutated Ago were unsuccessful, indicating that the DNA binding activity of Ago (which is likely preserved in its catalytic mutant) may be important for the inhibition of AgaP toxicity. Given the speculated nonspecific nuclease activity of AgaP, the plasmid resistance observed in cells expressing both AgaP and pAgo implies that plasmid DNA is more susceptible than chromosomal DNA to the Ago/AgaP system, perhaps due to their difference in copy number, chromatin‐like packing, and/or homologous repair efficiency.

It is worth noting that PLD superfamily proteins are widely distributed in bacteria, plants, and mammals and play important roles in various cellular functions. Previous studies have suggested that PLD is involved in plant immunity by generating a lipid mediator called phosphatidic acid and regulating hormone signaling^47^, ^48^, ^49^. In bacteria, the fusion of a N‐terminal PLD domain with a C‐terminal DNA‐recognition element presumably has given rise to a unique metal‐independent restriction enzyme known as BfiI^50^. Additionally, recent research has uncovered the involvement of a PLD domain‐containing protein in an antiviral process based on DNA phosphorothioation^41^. Taken together with our data, these findings suggest that participation in host immunity is a general trend among both eukaryotic and prokaryotic PLD proteins.

At last, we demonstrated that NpAgo alone could enhance gene editing efficiency in *E. coli* cells, and surprisingly, this effect was inhibited by the co‐expression of AgaP. We speculate that NpAgo improves gene editing by promoting homologous recombination of DNA substrates, which may be sterically hindered by its interaction with AgaP. This speculation is supported by our observation that a catalytically dead NpAgo alone provided low‐level immunity against the ssDNA virus (possibly by binding viral DNA and impeding its replication/transcription or by recruiting unknown host factors), which was, however, abolished in the presence of AgaP. Although our data showed that NpAgo‐assisted gene editing was independent of additive DNA guides, it cannot be excluded that NpAgo may load guides from the homology arms carried by the editing plasmid and then use them to target the chromosomal DNA.

In summary, our data uncovered the ability of catalytically active pAgo proteins to employ auxiliary proteins to strengthen immunity against different genetic invaders, highlighted the involvement of PLD proteins in Ago‐based host immunity, and exemplified the potential use of these pAgo proteins in genome editing in mesophilic organisms.

## MATERIALS AND METHODS

### Strains and culture conditions

Haloarchaeal strains used in this study are listed in Table S1. The *N. pellirubrum* DSM 15624 were grown at 37°C in nutrient‐rich AS‐168 medium (per liter, 200 g NaCl, 20 g MgSO_4_·7H_2_O, 3 g trisodium citrate, 2 g KCl, 1 g sodium glutamate, 50 mg FeSO_4_·7H_2_O, 0.36 mg MnCl_2_·4H_2_O, 5 g Bacto Casamino Acids, and 5 g yeast extract, pH 7.2). The uracil auxotrophic strain DF60 Δ*cas6* (*H. hispanica* ATCC 33960 Δ*pyrF* Δ*cas6*)^51^ strain or its derivatives were cultivated at 37°C in nutrient‐rich AS‐168 medium supplemented with uracil at a final concentration of 50 μg/ml. Strains transformed with the expression plasmid pWL502^52^ or its derivatives were grown in the yeast extract‐subtracted AS‐168 medium.

The *E. coli* DH5α strain was used for plasmid construction. For the investigation of the Ago‐assisted gene editing, the *E. coli* MG1655 strain was used. All *E. coli* strains were cultured in Luria‐Bertani (LB) medium at 37°C unless specified. When needed, antibiotics were added to the following concentrations: ampicillin (Amp), 100 μg/ml; kanamycin (Kan), 50 μg/ml; spectinomycin, 50 μg/ml; chloramphenicol, 25 μg/ml. When required, l‐arabinose was added to the medium at a final concentration of 10 mM to induce protein expression.

### Plasmid construction and transformation

The primers used are listed in Table S2. The *ago/agaP* operon or individual genes thereof were amplified from the *N. pellirubrum* genomic DNA by PCR using the high‐fidelity KOD‐Plus DNA polymerase (TOYOBO). The resulting DNA fragments were digested with restriction enzymes (New England Biolabs) and inserted into the predigested pWL502 backbone with T4 DNA ligase (New England Biolabs). For gene editing assay in *E. coli*, *ago* and *agaP* genes with codon optimized for *E. coli* expression were commercially synthesized (GenScript) and then cloned into expression vector under the control of l‐arabinose‐inducible P_*BAD*_ promoter. Overlap extension PCR was performed as previously described to introduce mutations^51^. All plasmids were verified by DNA sequencing. The transformation of the *H. hispanica* cells were performed according to the online Halohandbook (https://haloarchaea.com/wp-content/uploads/2018/10/Halohandbook_2009_v7.3mds.pdf). The yeast extract‐subtracted AS‐168 plates were used to screen the transformants. The transformation efficiency (colony forming unit per μg plasmid DNA, CFU/μg) was calculated based on three biological replicates.

As for *E. coli* cells, freezer stocks of *E. coli* were streaked on LB agar. The individual colonies were randomly picked and cultured overnight in 3 ml of liquid medium. The cultures were then subinoculated into a fresh medium in a ratio of 1:100 and grown to an OD_600_ of 0.4–0.6. The cells were pelleted by centrifugation and washed three times with ice‐cold 10% glycerol. Each pellet was resuspended in ice‐cold 10% glycerol. Electro‐transformation was performed using a MicroPulser Electroporator (Bio‐Rad) under appropriate settings (1.8 kV). The transformed cells were immediately suspended and recovered in 1 ml of LB medium before being spread onto LB agar plates containing appropriate antibiotics.

### Gene knock‐in

Gene knock‐in experiments for *H. hispanica* were performed according to previously described procedures^53^. To knock the *ago*/*agaP* operon into the *H. hispanica*, its DNA sequence and two fragments (~500 bp) located upstream and downstream of the *pyrF* (HAH_2085) on *H. hispanica* chromosome were separately amplified, then the resulting fragments were connected by overlap extension PCR. The linked fragment was digested and ligated into the suicide plasmid pHAR^53^. The plasmid was validated by DNA sequencing and then transformed into the *H. hispanica* cells. Construction of the *E. coli* MG1655 derivatives was performed using the CRISPR‐Cas9‐based system as previously described^54^. The mutants were validated by colony PCR and subsequent Sanger sequencing.

### RT‐PCR

A total of 3 ml of the late exponential culture of haloarchaeal cells was collected by centrifugation at 4°C. Total RNA was extracted using the TRIzol reagent (Invitrogen) according to the standard guidelines. A total of 20 µg of RNA was treated with DNase I (Thermo Fisher Scientific) according to the manufacturer's instructions to eliminate any contamination of DNA. RT reaction was performed using M‐MLV Reverse Transcriptase (Promega) and random hexamer primer (Thermo Fisher Scientific). Then, 1 μl of reverse transcripts was applied for PCR amplification using the corresponding primer pairs. The primers used for RT‐PCR are listed in Table S2.

### Virus interference assay

Three independent colonies were randomly selected and then inoculated into AS‐168 medium supplemented with uracil. The early‐stationary culture of *H. hispanica* cells was subinoculated into 10 ml of fresh medium for a 2‐day culturing. Two hundred microliters of the culture was mixed with 100 μl of 10‐fold serial dilutions of the HHPV‐2 virus and incubated for 30 min at room temperature. The mixture was then added to 3 ml of molten medium (0.7% agar) at 55°C and poured onto the prepared plates (1.2% agar). Once dried, the plates were incubated at 37°C for 3 days. The plaques were counted, and the plaque sizes were measured at 3 days post infection.

### Viral infection and growth curve measurement

For each strain, three individual colonies were randomly picked and independently cultured in AS‐168 liquid medium with uracil to the mid‐exponential phase. The optical density at 600 nm (OD_600_) of the cultures was monitored and the cell densities of the strains were adjusted to be equal before HHPV‐2 infection. A total of 500 μl of the exponential culture was mixed with the HHPV‐2 at an MOI of 10, the uninfected control was mixed with an equal volume of fresh medium, and the mixture was incubated at room temperature for 30 min. The samples were then inoculated into 100 ml of AS‐168 medium supplemented with uracil for culturing. The OD_600_ values were monitored every 12 h using the Shimadzu UV‐2550 spectrophotometer.

### Virus titration

To monitor the production of virus progeny, HHPV‐2‐infected cultures were collected at the indicated time points based on their growth curves. Samples were centrifuged at 4°C, and the pellets were removed. The supernatant was filtered (0.22 μm) and then serially 10‐fold diluted using the fresh medium, after which virus titers were estimated by spotting serial dilutions of samples on a lawn of *H. hispanica* cells. The PFUs were counted, and the mean and standard deviation were calculated from three replicates for each sample.

### Dilution spotting assay

To compare the growth of the WT, *ago* ^+^, *ago* ^+^/*agaP* ^+^, *ago* ^M^/*agaP* ^+^, or *ago* ^+^/*agaP* ^M^ *H. hispanica* cells harboring pWL502, we performed serial dilution spotting assay. The strains were cultured in yeast extract‐subtracted AS‐168 medium to the late exponential phase. The OD_600_ values of the cultures were adjusted to be equal to ensure their cell densities were almost equivalent. Five microliters of 10‐fold serial dilutions of the cultures were spotted on yeast extract‐subtracted AS‐168 plates or AS‐168 plates supplemented with uracil, and the plates were incubated at 37°C for 3–4 days.

### DNA preparation and whole‐genome resequencing

To determine whether APAP suppresses the intracellular propagation of the HHPV‐2 virus, whole‐genome resequencing was performed. According to the growth curve, *H. hispanica* cells were collected by centrifugation at specific time points. These cells were washed twice with fresh medium before DNA isolation. The genomic DNA of *H. hispanica* cells was extracted using the phenol‐chloroform method as previously described^55^. A total of 200 ng of genomic DNA was used to construct the sequencing library. The purified DNA was sonicated into 300–350 bp fragments. DNA library was constructed using the NEB Next® Ultra TM DNA Library Prep Kit according to the manufacturer's instructions. After treatment, DNA libraries were subjected to 150‐bp paired‐end sequencing on an Illumina Novaseq 6000 platform at GENEWIZ. The raw sequenced reads were trimmed to filter out low‐quality reads and remove adapters, after which the trimmed reads were analyzed.

### Gene‐editing assay

The empty vector pUC19 or derivates carrying *ago* or *ago/agaP* were separately introduced into the modified *E. coli* MG1655 cells. The resulting colonies were then separately cultured in LB medium with 10 mM l‐arabinose and then made competent for electro‐transformation. Aliquots (50 μl) of the electrocompetent cells were separately transformed with the mixture of 400 ng of editing plasmid with or without 1 μg of 5′ phosphorylated DNA guide (FW, or both FW and RV) targeting *gfp*. The shocked cells were recovered in 1 ml of LB medium containing 10 mM l‐arabinose for 1 h at 37°C. After subinoculation and a 10‐h cultivation, the cultures were separately plated on LB plates supplemented with kanamycin and l‐arabinose. After incubation for 15–24 h at 37°C, the number of surviving colonies was counted.

The plasmids carrying *ago* or *ago/agaP* were separately co‐transformed with editing plasmid into *E. coli* cells. For each experimental setting, three individual colonies were randomly picked and cultured in an LB liquid medium containing ampicillin (100 μg/ml), chloramphenicol (25 μg/ml), and l‐arabinose (10 mM) for 10 h. The cultures were then sub‐inoculated into fresh medium supplemented with ampicillin, chloramphenicol, and l‐arabinose for another 10‐h culturing. After cultivation for three passages at 37°C, the culture from the final passage was sampled, serially diluted, and aliquots of samples were plated onto the LB medium supplemented with ampicillin, chloramphenicol, and l‐arabinose (nonselective medium) or the LB medium supplemented with kanamycin and l‐arabinose (selective medium). Then, the plates were incubated for 15–24 h at 37°C after which the cells were counted. The survival ratio was calculated by dividing the number of colonies on the selective medium by the number of colonies on the nonselective medium. The kanamycin‐resistant colonies were validated by colony PCR using specific primers (seq‐F and seq‐R in Table S2), and the PCR products were subjected to DNA sequencing with the primer kanS‐F (Table S2).

### Sequence and structure analysis

Amino acid sequences were aligned and viewed using ESPript v3.0. For structure analysis, structures from Nuc (PDB: 1BYR) were loaded in PyMOL. The domain or residue alignments were analyzed using the PyMOL software. The phylogenetic tree was generated by the maximum likelihood method using MEGA X software and visualized using iToL v5^56^.

### Data analysis and image visualization

The data were plotted and analyzed using GraphPad Prism version 8.0 and Microsoft Excel, respectively. *p* < 0.05 was considered statistically significant.

## AUTHOR CONTRIBUTIONS

**Feiyue Cheng**: Conceptualization (equal); formal analysis (lead); funding acquisition (equal); investigation (lead); visualization (equal); writing—original draft (lead); writing—review and editing (equal). **Aici Wu**: Formal analysis (equal); investigation (equal); visualization (equal). **Zhihua Li**: Investigation (supporting). **Jing Xu**: Formal analysis (supporting). **Xifeng Cao**: Investigation (supporting). **Haiying Yu**: Formal analysis (supporting). **Zhenquan Liu**: Investigation (supporting). **Rui Wang**: Funding acquisition (supporting); investigation (supporting). **Wenyuan Han**: Funding acquisition (supporting); writing—review and editing (supporting). **Hua Xiang**: Conceptualization (equal); formal analysis (equal); funding acquisition (lead); project administration (equal); writing—review and editing (equal). **Ming Li**: Conceptualization (lead); formal analysis (equal); funding acquisition (equal); project administration (lead); visualization (lead); writing—original draft (equal); writing—review and editing (lead).

## ETHICS STATEMENT

No animal or human experiments were involved in this study.

## CONFLICT OF INTERESTS

Ming Li, Feiyue Cheng, Aici Wu, and Zhihua Li have filed a related patent.

## Supporting information

Supporting information.

## Data Availability

The DNA sequencing data were deposited in the National Microbiology Data Center (NMDC) (https://nmdc.cn/resource/genomics/project) with accession number NMDC10018626.
