# Supplementary material for: Catalytically active prokaryotic Argonautes employ phospholipase D family proteins to strengthen immunity against different genetic invaders
Source: mLife. 2024 Sep 4;3(3):403–16. doi: 10.1002/mlf2.12138 (PMC11442185; doi:10.1002/mlf2.12138)
Supplement: Supplementary file 1 — Supporting information. [file MLF2-3-403-s001.docx]

**Catalytically active prokaryotic Argonautes employ phospholipase D family proteins to strengthen immunity against different genetic invaders**

Feiyue Cheng ^#,*^, Aici Wu ^#^ Zhihua Li, Jing Xu, Xifeng Cao, Haiying Yu, Zhenquan Liu, Rui Wang, Wenyuan Han, Hua Xiang^*^, Ming Li^*^

**The file includes:**

Figures S1 to S5

Tables S1 to S2

References


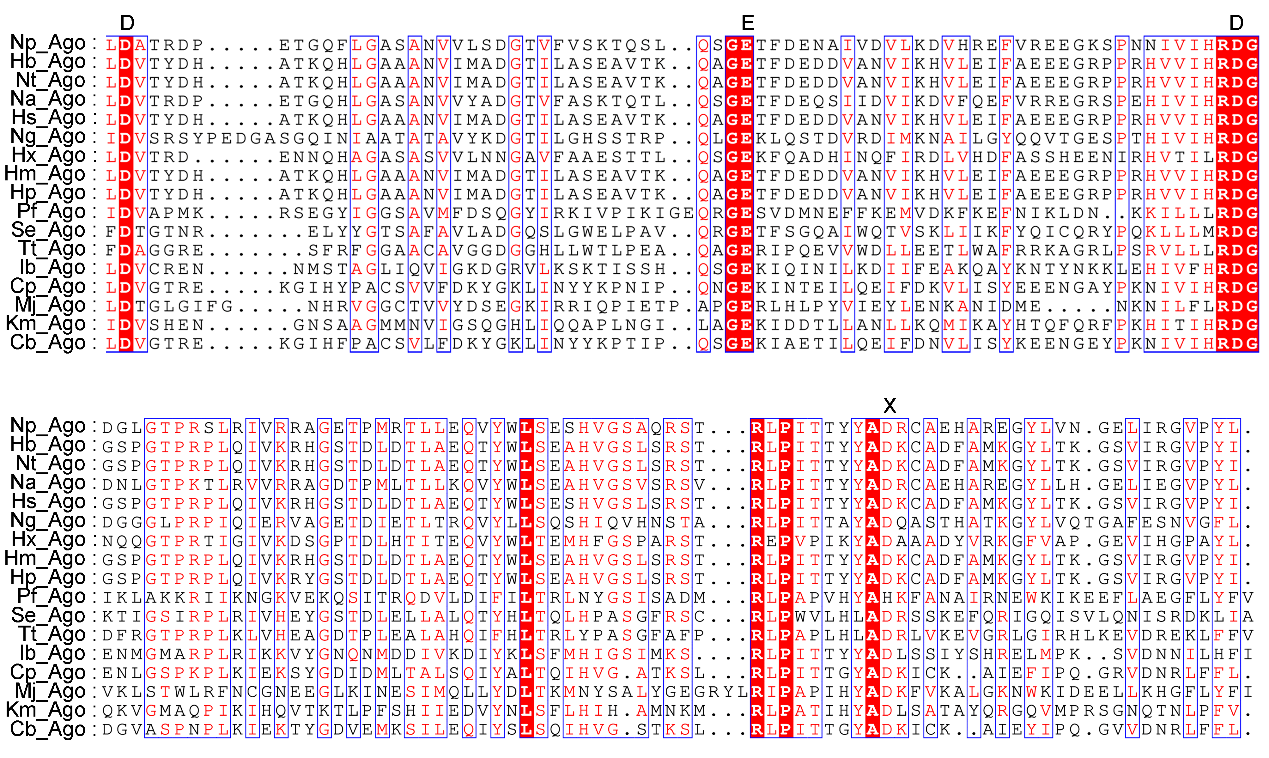


Figure S1. Sequence alignment of Argonaute proteins from different prokaryotes. The conserved DEDX catalytic residues are shown. Np, Natrinema pellirubrum DSM 15624; Hb, *Halogeometricum borinquense* DSM 11551; Nt, *Natronorubrum tibetense* GA33; Na, *Natrialba asiatica* DSM 12278; Hs, *Halosimplex salinum* YPL4; Ng, *Natronobacterium gregoryi* SP2; Hx, *Halopiger xanaduensis* SH-6; Hm, *Halorussus marinus* LYG-36; Hp, *Haloferax profundi* SB29; Pf, *Pyrococcus furiosus* DSM 3638; Se, *Synechococcus elongatus* PCC 6311; Tt, *Thermus thermophilus* SG0.5JP17-16; Ib, *Intestinibacter bartlettii* DSM 16795; Cp, *Clostridium perfringens* WAL-14572; Mj, *Methanocaldococcus jannaschii* DSM 2661; Km, *Kurthia massiliensis* JC30; Cb, *Clostridium butyricum* CBM588.


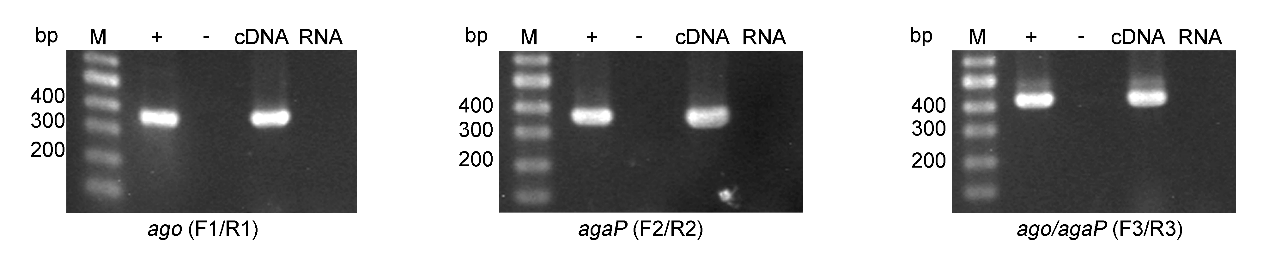


Figure S2. RT-PCR determination of the transcription pattern of the *N. pellirubrum* *ago-agaP* operon in *H. hispanica*. +, positive control (genomic DNA); -, negative control (no templates); RNA, the DNA-digsted RNA samples; cDNA, the complementary DNA reverse transcribed from DNA-free RNA samples using random primers (see Materials and Methods). The primers used for each assay are indicated below the corresponding gel. M, dsDNA size marker.


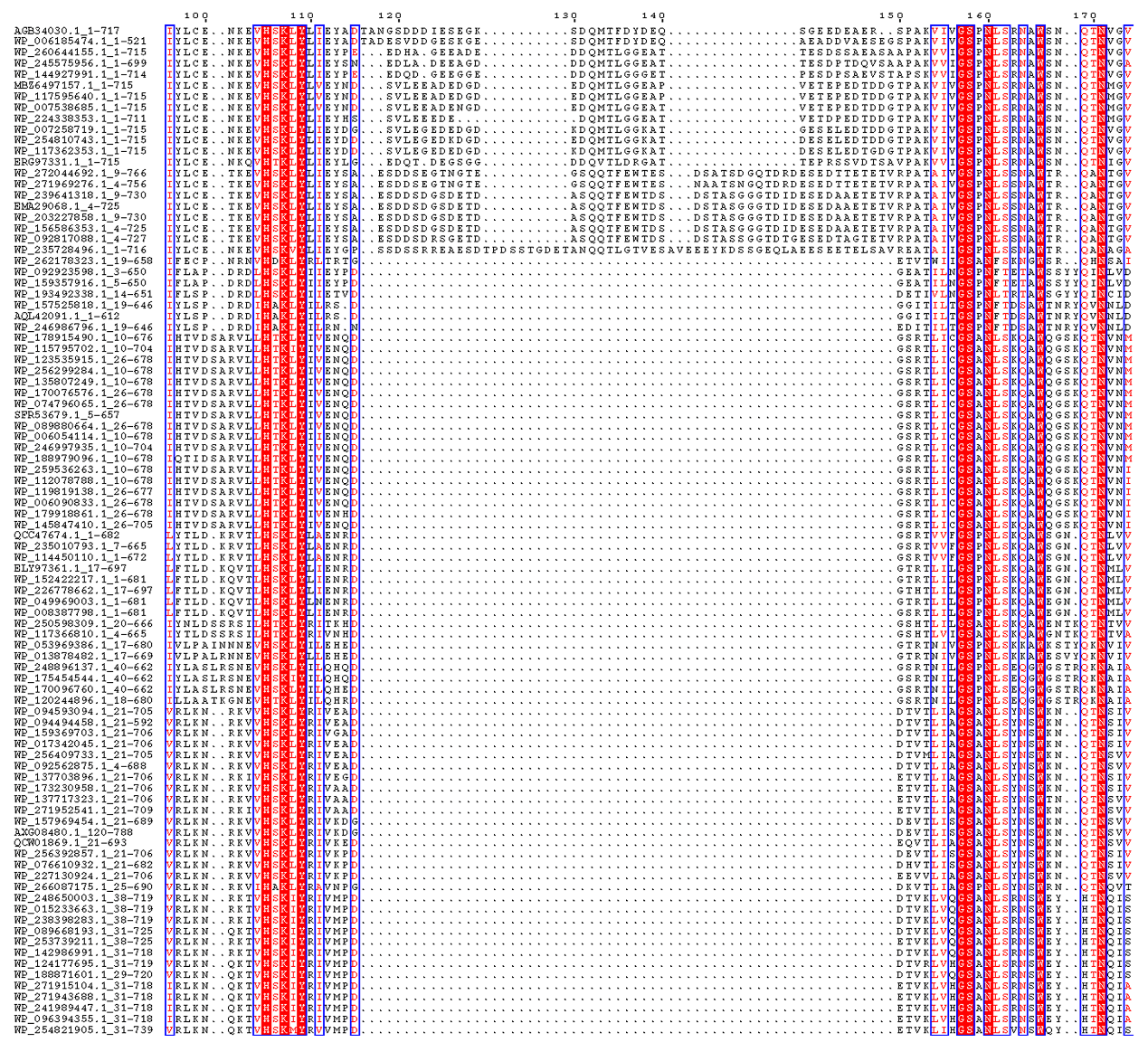


Figure S3. Sequence alignment of *N. pellirubrum* AgaP and its most related homologs. The catalytic residues ‘HxK’ and ‘GSxN’ are highly conserved.


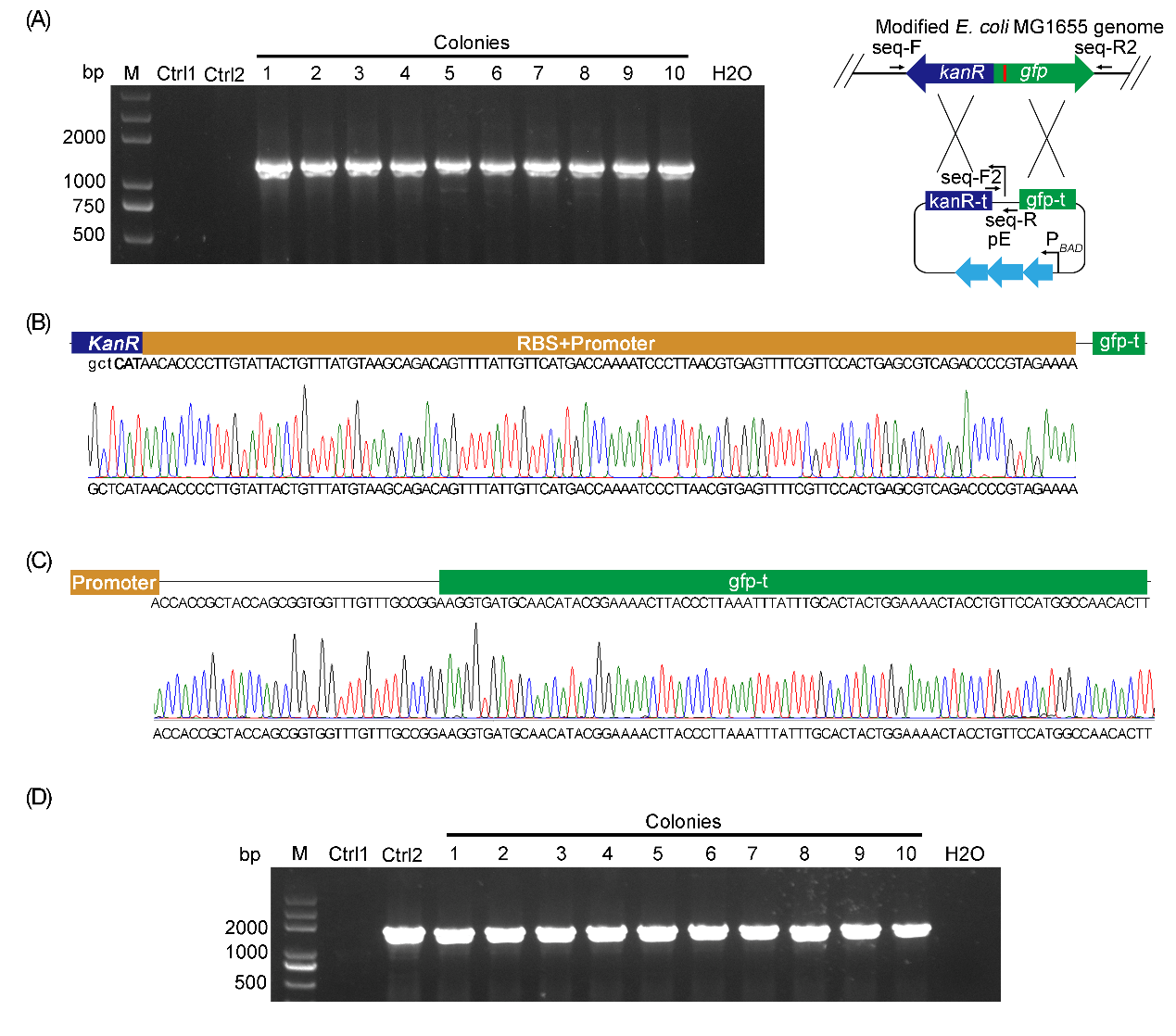


Figure S4. NpAgo-assisted gene-editing cells contained the designed promoter sequences. (A) Ten kanamycin-resistant survivors from the NpAgo-assisted editing were randomly selected for PCR analysis using the primer pair seq-F/seq-R. Lane M, dsDNA size marker. Ctrl1 and Ctrl2 respectively indicate the wild-type and modified MG1655 cells containing the editing plasmid. (B) Validation of the genotype of kanamycin-resistant colonies by DNA sequencing. The representative sequencing result was shown. (C) Primers seq-F2 and seq-R2 were also used for PCR analysis of the kanamycin-resistant survivors, and the PCR products were subjected to DNA sequencing with primer gfpS-R (Table S2). (D) Ten kanamycin-resistant survivors after recombination were selected for PCR analysis using the plasmid backbone-specific primer pair. Ctrl1, negative control. Ctrl2, positive control.


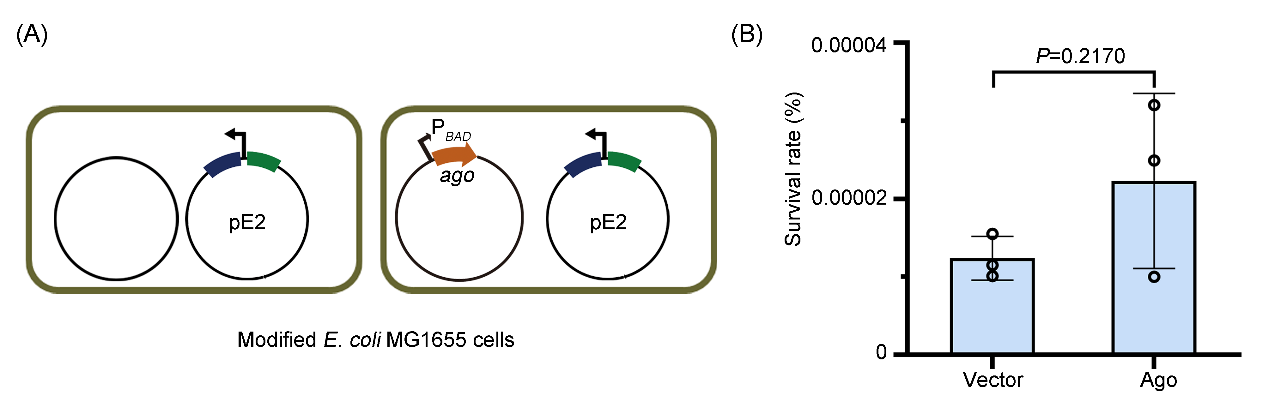


Figure S5. Evaluation of the effect of NpAgo in the absence of λ-Red system. (A) *E. coli* cells were co-transformed by pE2 (without λ-Red system) and a plasmid carrying *ago* (or the empty vector). (B) The survival rate of *E. coli* cells. Error bars, mean ± s.d. (n=3).

Table S1 Strains used in this study

| **Strain Name** | **Description** | **References** |
| --- | --- | --- |
| *E. coli* MG1655 | Wild-type *E. coli* K-12 | Lab collection |
| *E. coli* MG1655 Δ*lacZ*::*gfp*-*kanR* | Modified *E. coli* MG1655 with *lacZ* deletion, carrying a *gfp* gene and a promoter-lacking *kanR* gene | This study |
| WT | *∆pyrF ∆cas6* mutant of *H. hispanica* ATCC 33960 | (1) |
| *ago*+ | Modified WT with *N. pellirubrum* *ago* | This study |
| *ago*+/*agaP*+ | Modified WT with *N. pellirubrum* *ago-agaP* | This study |
| *ago*+/*agaP*^M^ | Modified *ago*+/*agaP*+ with *agaP* mutated (H105A/K107A) | This study |
| *ago*^M^/*agaP*+ | Modified *ago*+/*agaP*+ with *ago* mutated (D630A) | This study |
| *ago*^M^ | Modified *ago*+ with the catalytic residue D630 mutated to alanine | This study |

Table S2 Oligonucleotides used in this study

| **Name** | **Sequence (5´-3´)^a^** | **Description** |
| --- | --- | --- |
| **For amplification of *ago/agaP*, *ago* or *agaP*** | | |
| AP-F | CGCGGATCCAGGGACATTTGAAACACGCTA | Forward primer for *ago* or *ago*/*agaP* amplification |
| AP-R | CGGGGTACCCGGCTAACGAATAACTGATGGT | Reverse primer for *ago*/*agaP* amplification |
| Ago-R | CGGGGTACCGCTGTCTCAGAGGTATGGGA | Reverse primer for *ago* amplification |
| AgaP-F | ATGCAATTCGACATTCCGATT | Forward primer for *agaP* amplification |
| Pnat-R | AATCGGAATGTCGAATTGCATCGATTCGAACGAGATGGCCGTA | Linking the native promoter to the *agaP* gene |
| **For reverse transcription-PCR** | | |
| F1 | CTCGACGACAGGTGGATTCT | against the sequence of *ago* |
| R1 | CGTCGAACGGTTCGAGGAT | against the sequence of *ago* |
| F2 | GATCGCCGGCGAAAGTCAT | against the sequence of *agaP* |
| R2 | GGATTCATCGCTCGATGAT | against the sequence of *agaP* |
| F3 | GGAGCAGGTGTACTGGCTAA | against the sequence of *ago* |
| R3 | GTCACCGACGATCACCTCAA | against the sequence of *agaP* |
| **For gene knock-in** | | |
| Up-F | CGGGGTACCCGACTCGGCTCGGCAATA | Amplifying the upstream sequence of *pyrF* gene |
| Up-R | TAGCGTGTTTCAAATGTCCCTCGGCTGGCAGCGATACAA | Amplifying the upstream sequence of *pyrF* gene |
| Down-F | ACCATCAGTTATTCGTTAGCCGAAACAGCTCAAACAGCGACTG | Amplifying the downstream sequence of *pyrF* gene |
| Down-R | CCCAAGCTTCCAGCATTCCGAGTATCCAGAG | Amplifying the downstream sequence of *pyrF* gene |
| **For *agaP* mutation** | | |
| H105-F | GAGAATAAGGAAGTCGCCTCCAAACTCTATCTG | H105A |
| H105-R | GGAGGCGACTTCCTTATTCTCGCAGAG | H105A |
| K107-F | AAGGAAGTCCACTCCGCACTCTATCTGATCGAG | K107A |
| K107-R | GAGTGCGGAGTGGACTTCCTTATTCTC | K107A |
| E112-F | AAACTCTATCTGATCGCGTATGCAGACACCGCT | E112A |
| E112-R | ATACGCGATCAGATAGAGTTTGGAGTG | E112A |
| D115-F | CTGATCGAGTATGCAGCCACCGCTAATGGGTCT | D115A |
| D115-R | GGTGGCTGCATACTCGATCAGATAGAGTTTG | D115A |
| G119-F | GCAGACACCGCTAATGCGTCTGATGACGATATC | G119A |
| G119-R | AGACGCATTAGCGGTGTCTGCATACTC | G119A |
| S120-F | GACACCGCTAATGGGGCTGATGACGATATCGAG | S120 |
| S120-R | ATCAGCCCCATTAGCGGTGTCTGCATAC | S120A |
| G157-F | GCGAAAGTCATCGTCGCTTCTCCGAACCTCTCA | G157 |
| G157-R | AGAAGCGACGATGACTTTCGCCGGCGA | G157A |
| S158-F | AAAGTCATCGTCGGTGCTCCGAACCTCTCACGC | S158 |
| S158-R | CGGAGCACCGACGATGACTTTCGCCGG | S158A |
| N160-F | ATCGTCGGTTCTCCGGCCCTCTCACGCAATGCG | N160A |
| N160-R | GAGGGCCGGAGAACCGACGATGACTTTC | N160A |
| **For *ago* mutation** | | |
| D630-F | CTGCTTCGTCGGCCTCGCTGCGACACGCGACCCGG | D630A |
| D630-R | CCGGGTCGCGTGTCGCAGCGAGGCCGACGAAGCAG | D630A |
| mYK-UR | TTGCTTGCCGAGCGATTTCGCGAACTCGGGGGCCGGATCGTCGATGGTCCC | Y570A, K574A |
| mYK-DF | GGGACCATCGACGATCCGGCCCCCGAGTTCGCGAAATCGCTCGGCAAGCAA | Y570A, K574A |
| **NpAgo-assisted gene editing assay** | | |
| PBAD-F | GAGTGAGCTGATACCGCTCGAAGCTTCATCGATTTATTATGACAAC | For amplification of P*_BAD_* promoter |
| PBAD-R | CATTTTTTATAACCTCCTTAGAGC | For amplification of P*_BAD_* promoter |
| Eago-F | CTCTAAGGAGGTTATAAAAAATGCCGACCCAGAGCGACATTG | For amplification of codon-optimized *ago* gene |
| Eago-R | ACGGTGAAGAATTCGAGCTCGGTACCTTACAGATACGGAACACCAC | For amplification of codon-optimized *ago* gene |
| EagaP-F | CTCTAAGGAGGTTATAAAAAATGCAGTTCGACATCCCGATTG | For amplification of codon-optimized *agaP* gene |
| EagaP-R | ACGGTGAAGAATTCGAGCTCGGTACCTTAACGCTTCAGAATGCTAC | For amplification of codon-optimized *agaP* gene |
| pTF-F | AGTCCTAGGTATAATACTAGTCACGCCGCATCCAGCGCTGAGTTTTAGAGCTAGAAATAGCA | For amplification of *lacZ*-targeting gRNA |
| pTF-R | ACTAGTATTATACCTAGGACT | For amplification of *lacZ*-targeting gRNA |
| Ins-F | CTTTTTTTGAATTCTCTAGAGTCGACGACCAGACACCCATCAACAG | For amplification of sequence of insertion fragment |
| Ins-R | GGATAACAGGGTAATAGATCTAAGCTTGGTGAACATGATGCCGACAAT | For amplification of sequence of insertion fragment |
| seq-F | GCGGATATCTCGGTAGTGGGATACG | For PCR screening of kanamycin-resistant colonies |
| seq-R | CCGGCAAACAAACCACCGCTGG | For PCR screening of kanamycin-resistant colonies |
| kanS-F | GTTCAACAGGCCAGCCATTACGCTC | For DNA sequencing |
| seq-F2 | AAGATCAAAGGATCTTCTTGAGATC | For PCR analysis of kanamycin-resistant colonies |
| seq-R2 | GAACGGCGCAAACATCACTAACATGC | For PCR analysis of kanamycin-resistant colonies |
| gfpS-R | GTCTGCCATGATGTATACATTG | For DNA sequencing |
| pE-tesF | CTGCCTTAAAAAAATTACGCCCCGC | For PCR analysis of kanamycin-resistant colonies |
| pE-tesR | GGAACTGAGTGTCAGGCGTGGAATG | For PCR analysis of kanamycin-resistant colonies |
| FW | TTAAAGAAGATGGAAACATTCTTG | Forward guide DNA |
| RV | CAAGAATGTTTCCATCTTCTTTAA | Reverse guide DNA |

^a^underlined, restriction sites; F, forward primer; R, reverse primer.

**REFERENCES**

1. Li M, Gong LY, Cheng FY, Yu HY, Zhao DH, Wang R, et al. Toxin-antitoxin RNA pairs safeguard CRISPR-Cas systems. *Science*. 2021;372:eabe5601.
